# Perception of Organic Food Consumption in Romania

**DOI:** 10.3390/foods6060042

**Published:** 2017-05-30

**Authors:** Anca Gabriela Petrescu, Ionica Oncioiu, Marius Petrescu

**Affiliations:** 1 Faculty of Economic Sciences, Valahia University, Carol I Bvd, No.2, Targoviste 130024, Romania; 2 Faculty of Banking and Finance, Accountancy and Business Administration, Titu Maiorescu University, Calea Vacaresti Street, No.189, Bucharest 040051, Romania; nelly_oncioiu@yahoo.com; 3 Faculty of Economic Sciences, Valahia University, Carol I Bvd, No.2, Targoviste 130024, Romania; petrescu.marius_m@yahoo.com

**Keywords:** sustainable consumption, consumer behavior, organic foods, food security

## Abstract

This study provides insight into the attitude of Romanian consumers towards organic food. Furthermore, it examines the sustainable food production system in Romania from the perspective of consumer behavior. This study used a mathematical model of linear regression with the main purpose being to determine the best prediction for the dependent variable when given a number of new values for the independent variable. This empirical research is based on a survey with a sample of 672 consumers, which uses a questionnaire to analyze their intentions towards sustainable food products. The results indicate that a more positive attitude of consumers towards organic food products will further strengthen their purchasing intentions, while the status of the consumption of organic consumers will not affect their willingness to purchase organic food products. Statistics have shown that sustainable food consumption is beneficial for health, so it can also become a profitable business in Romania. Furthermore, food sustainability in Romania depends on the ability of an organic food business to adapt to the new requirements of green consumption.

## 1. Introduction

Malnourishment, malnutrition, and food insecurity are the major challenges that have led to the consideration of overall sustainability [1]. In the medium- and long-term, there are strong variations recorded at regional levels regarding the traditional and organic foods produced globally [2]. 

Generally, food quality is a major concern because it directly affects the health of the population. It is known that certain deficient consumption patterns are associated with four to ten causes of death (coronary heart disease, some cancers, stroke, or type 2 diabetes). Thus, organic food is considered as a means of improving the overall health of the population [3].

The question now is whether organic small businesses are economically sustainable. Organic small businesses only represent a small proportion of the total food market (around 1–3%), but have significant potential for growth in the near future [4]. Growing consumer demand leads to rapidly increasing new market opportunities for organic food producers, retailers, and distributors [5]. According to Yussefi and Willer [6], all organic agricultural land worldwide takes up around 24 million hectares (633,891 farms). Organic farming uses 11% of the total area in Austria, 10% in Switzerland, 8% in Italy, 7% in Finland, around 6% in Denmark and Sweden, and 4% in Germany. However, it is only between 1 and 2% in many countries, such as Romania, Australia, Argentina or France, as well as being below 1% in USA or Brazil. 

Most consumers are convinced that organic products are more nutritious than non-organic products. However, organic products are affected by numerous factors, including the geographical location of the farm, local soil characteristics, climatic conditions that can vary from one growing season to another, maturity at the time and storage of harvest, as well as the time of testing after harvest [7,8]. In addition, there are concerns regarding mislabeling practices by attaching trademarks to food only described as organic [9].

The demand for growth depends on the strength of consumers’ trust in the organic supply chain and the organic producer [10]. A previous study [11] has described how the buying patterns of consumers follow a fairly predictable path, as the main organic products that consumers typically buy are dairy products, non-dairy products (such as soy milk), and baby food. The second group of products usually encompasses juices, single-serve beverages, meat/poultry, cold cereals, and snacks.

Ajzen and Fishbein [12] tried to estimate the discrepancy between attitude and behavior by using the theory of planned behavior. According to the theory, the individual’s intention to perform a behavior is a combination of their attitude towards performing the behavior and consideration of subjective norms. It also states that only specific attitudes toward the behavior in question can be expected to predict that behavior. In any case, the authors consider their theory of reasoned action as a process model for the prediction of various kinds of behavioral choices. 

The current consumers of organic food are diverse, but most studies characterized the organic consumer as Caucasian, affluent, and well-educated, in addition to being concerned about health and product quality [13]. The key message of Chen’s study [14] is that there is a strong significant relationship between health consciousness and attitude towards purchasing organic food. He explains that the environmental consciousness of consumers encourages them to have a positive attitude towards the purchase of organic food. This is consistent with another study [15], which found that buying organic foods depends on the respect of three essential principles: health consciousness, the equilibrium of the economic situation, and the protection of the environment.

At the same time, the average age of organic consumers is clustered in two age groups: 18–29 years old and 40–49 years old [16]. Tarkiainen and Sundqvist [17] aimed to widen the current perspective on the effect of subjective norms on attitude and intention to purchase organic food. The subjective norms are defined as the social pressure for an individual to engage or comply with a group behavior, such as family and friends. Following the view that there are gender differences in attitude and intentions, they argue that different genders will also provide different behavioral intentions. A more recent study [18] has shown that the variables affecting the attitudes of consumers towards buying organic food affect their intentions to purchase. He indicates that male and female consumers have different attitudes and intentions to buy organic food, which thus confirms the findings of previous studies that gender affects the purchasing behavior of organic food.

Since 2005, over 90% of Romanian bio production has been exported to countries such as the UK, Italy, and Germany. In addition, Romanian behavior has started to be characterized by modern human consumption behavior [19]. 

In addition, the income of Romanian consumers and the price of products represent major determinants of food demand in the classical economic theory. This relationship is assumed to be significant—especially in low-income groups. Given the large proportion of low-income areas in Romania, one assumes that the importance of these factors is and shall remain significant in the near future in Romania. 

There are three main objectives of the current study. First, it examines the influence of social and personality factors on the attitudes of Romanian consumers towards organic foods. Second, it investigates the relationship between consumer attitudes and purchase intention of organic foods. Third, it examines the influence of social and personality factors on the purchase intention of organic products. Based on these observations, the producers and marketers of organic foods can come up with different strategies to engage consumers and encourage them to buy organic products. 

Essentially, rural Romanian farms are a reality of sustainable agriculture. Because these farms produce organic food, they have a social role and will continue to exist by selling certain quantities of organic exports. Therefore, the authors of this paper try to answer the questions: How do the beliefs of Romanian consumers influence their attitude and intention to purchase organic food products? What is the motivational reality of Romanian consumers’ behavior behind the purchase of organic food? What are the perceptions of Romanian consumers regarding sustainable food? Are there really essential effects on our health when eating organic foods?

In spite of the current difficulties, the conducted survey has shown interesting elements that could considerably modify the outlook of the organic foods market in the future. The results of this research should stimulate small entrepreneurships of organic foods to create strong brand images in the minds of Romanian consumers for each product. The ability to come up with new solutions will therefore depend on the entrepreneurship ability of Romanians to adapt their resources in response to the new requirements of the green consumption.

## 2. Literature Review and Development of Hypotheses

In the literature, organic food has been approached from several points of view, although only three of these are significant to this present study. There is a widespread belief that organic food is substantially healthier and safer than conventional food, with consumers willing to pay significant price premiums to obtain a debt ratio to converge back to its initial level [20]. A second specification—used by a previous study [21]—also proposes tougher restrictions for the present nutritional value, as the quality of organically grown food has provided only isolated results regarding the safety characteristics of the produce. There is an important body of literature discussing the psychology of the resistance against change and against organic food [22,23,24]. Each study design has several advantages and disadvantages. Furthermore, White and Duram [25] require a series of nutritional recommendations to limit conventional consumption.

In this context, addressing the question of the safety of organic produce becomes of major importance, as recent statistics have shown that organic food consumption is aggregately still low in proportion, compared to non-organic food [26].

Ample studies have been undertaken during the last decade with respect to the attitude and intention of consumers towards purchasing organic food—especially in developing countries where it is a real challenge to sell organic foods [6,27]. We should not forget the importance of food security, which results in three specific goals: ensuring adequate food production; maximizing the stability of the supply flow of agricultural products, and providing access to available agricultural resources for those who need them. In essence, we must ensure that basic foods which are necessary for human health are provided [28].

In addition, another study [29] has discussed that the emphasis is on green marketing issues—especially with regards to the demand of organic food consumers. Profiling of organic consumers has not made it clear whether consumers consider sustainable products as being important for their health or whether the structure of calorie consumption ensures good health. Clarifying these uncertainties may determine a new approach regarding organic food. In response to the consumption–sustainability dilemma, organic food could be an initiative for decoupling negative environmental impacts from the actual economy, especially in food-related areas where the consumers see health benefits.

On the other hand, food security in most developing countries depends in part on the sustainable use of natural resources and the sustainable provision of ecosystem services. The ecosystem functions directly and indirectly to influence each of the dimensions of food security through the provision of ecosystem services that support agricultural production, create income-generating opportunities, and provide energy for cooking. Thus, sustaining these functions is crucial for ensuring global food security. A food security strategy must incorporate the fact that production, income, market stability, food prices, and social safety schemes are all interdependent [30,31]. In a different view, Huang, Lee, and Ho [32] came to the conclusion that attitudes cannot be observed directly and researchers must rely on determining consumer attitudes through measurements. With regard to consumers, attitudes towards organic food products can be influenced by a number of antecedents. An evaluation will impact the judgement of an individual’s attitude.

Social factors clearly play a strong role, including the normative and informational susceptibility towards social influence. Social influence is reflected in the effect of others’ judgment on the behavior of an individual consumer. The two different forms of consumer susceptibility are information susceptibility and normative susceptibility. Information susceptibility refers to the purchasing decisions made by consumers based on the expert opinions of others, while a normative susceptible person might make a decision based on the expectations of what would impress others [33]. Individuals tend to remain true to their inner values, and will tend to form attitudes that have influences on consumers’ attitudes towards purchasing organic food. For the present research, we formulate the following hypotheses:

**Hypothesis 1** (H1)**.** Social factors have an influence on the attitudes of consumers towards the purchase of organic food.

There is a significant relationship between attitudes towards organic foods and purchase intention. The query theory [34] is a framework that incorporates attentional processes and memory-retrieval operations. It assumes that people—when asked to delay consumption—first assess the evidence arguing for immediate consumption and only then assess evidence that argues for delaying consumption. According to the theory of planned behavior, purchase behavior is determined by purchase intentions, while the purchase intention is in turn determined by attitudes [35]. Instead of attitudes towards the product, attitudes towards behavior are important in better predicting behavior [21,26]. Furthermore, attitudes towards organic foods have an important positive influence on purchase intention [32,36]. More specifically, attitudes measure one’s evaluation about the perceived behavioral control of organic food, while the subjective norm measures the degree to which individuals are driven solely by what others think they should do.

**Hypothesis 2** (H2)**.** Organic food prices affect the attitude towards the purchase of organic food.

The prices of products are valued not as much for reflecting the functional quality, but more for their ability to reflect an individual’s status. A previous study [37] extensively examined the status of consumption in consumers looking for self-satisfaction in addition to wishing to display their prestige and status in front of others. In other words, consumers are stimulated to be more willing to buy and can even pay a higher value for products with status. There are situations when the prices are increased in order to take advantage of people seeking healthy choices. Thompson [38] rightfully concluded that it is important to analyze the price of organic foods along with comparable conventional foods in order to find the economic factors that influence each consumer segment.

Other factors noted include the helpfulness in reviews regarding bioenergy value and credibility. The levels of prior knowledge about and interest in organic food products can vary among consumer profiles. Review valuations may be more stable for some consumers than others [39,40,41]. 

## 3. Research Methods

The primary data of this research were gathered from a questionnaire distributed online for Republica BIO consumers [42], which was developed based on the nine-point Likert-scale adopted from Ajzen [35]. There are four sections. Sections A and B of the questionnaire measured social factors. Section C evaluated attitudes and purchase intentions of organic foods. Section D contained items regarding the influence of gender and age of respondents. All items were measured on a seven-point Likert-scale with one representing “I totally disagree” to seven representing “I totally agree”. 

Table 1 presents the description of scale items, with reflections of their validities and reliabilities.

This research used a quantitative approach with the quota-purposive and convenience sampling methods. The experiment was conducted from May 2016 until October 2016, with the target population being Romanian organic food consumers. A sample of 723 responses was obtained. However, the number of responses was reduced because some respondents failed to answer the questionnaire completely. A total of 672 responses were considered acceptable after the reliability and validity was tested. These responses were then entered into SPSS 20.0 to be calculated using path analysis with an approach to regression analysis for hypothesis testing. The instruments were tested by conducting validity, reliability, and normality tests. The age range of the participants was between 18 and 50 years. Table 2 provides the sample descriptive characteristics.

The authors propose the development of a linear model similar to:

(1) $$y=\beta_{0}+\beta_{1x1}+\beta_{2x2}+\ldots +\beta_{6x6}+\varepsilon$$

linking *y* to the seven explanatory variables from Table 1.

Starting from the input data, we wish to estimate the parameters $\beta_{0},\beta_{1},\ldots ,\beta_{p}$ (in our case, *p* = 7). We are using the “least squares” method to minimize the sum of squared errors. Note that the error is:

(2) $$\varepsilon_{i}=y_{i}-\beta_{0}-\beta_{1xi1}-,\ldots ,-\beta_{pxip}$$

Additionally, the sum of squared errors is:

(3) $$s\left(\beta_{0},\beta_{1},\ldots ,\beta_{p}\right)=\overset{n}{\underset{i=1}{\sum }}\varepsilon_{1}^{2}{\left(y_{1}-\beta_{0}-\beta_{1xi1},\ldots ,-\beta_{pxip}\right)}^{2}$$

By derivation in relation to each of the *p* + 1 variables and equaling this result to zero, we obtained a linear system whose solution ${\hat{\beta }}_{0},{\hat{\beta }}_{1},\ldots ,{\hat{\beta }}_{p}$ minimizes $s\left(\beta_{0},\beta_{1},\ldots ,\beta_{p}\right)$.

The estimated value ${\hat{\beta }}_{0}$ is called the intercept or constant, while ${\hat{\beta }}_{j}$ is the regression c. By exploiting the estimated regression coefficients ${\hat{\beta }}_{0},{\hat{\beta }}_{1},\ldots ,{\hat{\beta }}_{p}$, we are able to write the linear regression equation as follows:

(4) $$\hat{y}={\hat{\beta }}_{0}+{\hat{\beta }}_{1x1}+,\ldots ,+{\hat{\beta }}_{pxp}$$

For each observation from our data, we can calculate:

(1) The variables predicted by the model:
  (5) $${\hat{y}}_{i}={\hat{\beta }}_{0}+{\hat{\beta }}_{1xi1}+,\ldots ,+{\hat{\beta }}_{pxip}$$
(2) The corresponding residuals:
  (6) $${\hat{e}}_{i}=y_{i}-{\hat{y}}_{i},\;i=1,2,\ldots ,n$$
(3) Advance the squares sum of the residual numbers:
  (7) $$sse=\overset{n}{\underset{i=1}{\sum }}{\left(y_{i}-{\hat{y}}_{i}\right)}^{2}=\overset{n}{\underset{i=1}{\sum }}ei^{2}$$

where the coefficients estimate the $y_{i}$ predicator.

In order to test this hypothesis, the “chi square” method will be used, which determines whether there is any significant difference between the expected and observed frequencies that are specific to this variable. The use of such a statistical method can determine the identification of two hypotheses. The first one (the null hypothesis) can show us that there are no important differences between the two types of frequencies expected and observed associated to the variables analyzed in this article. In comparison, the second hypothesis (the alternative hypothesis) can identify a negation of the null hypothesis.

The main advantage in analysis to using this method is the use of the SPSS 20.0 package of applications (SPSS Inc., Chicago, IL, USA). This package allows for the opportunity to configure “cross-tab” tables after identifying, receiving, and analyzing statistical information obtained from the research in the program database.

## 4. Results and Discussions

The interpretation of the results is under effect of the scope of the research, which regards the characteristics of the chosen cases and the approach of the consumer side.

The sample characteristics showed a larger number of women (59.23%) as compared to men (40.77%). Findings also indicated that the relationship between gender and positive attitudes towards organic foods was mediated by (1) perceptions of the positive effect on consumer attitudes towards the purchase of organic food, (2) influences of gender and age on the health consciousness of consumers, and (3) traditional attitudes toward organic foods related to the probability of individuals paying more for this type of product.

The reliability is showed by the value of alpha, with a value of 0.600 considered reliable. The results of the reliability analysis using SPSS software (particularly with regards to the path coefficient) are presented in Table 3, which shows that social factors have a positive effect on attitudes towards organic foods.

This can be seen from the result showing that the relationship between social factors and attitudes towards organic foods is positive (0.251) and significant (<0.05). Furthermore, the path coefficient between novelty seeking and attitudes towards organic foods is positive (0.322) and significant (<0.05), and is thus consistent with hypothesis 1.

The path coefficient between quality and attitudes towards organic foods is negative (−0.278) and significant (<0.05). As such, hypothesis 1 was supported by the path coefficient between personal gratification and attitudes towards organic foods, which was found to be negative (−0.235) and significant (<0.05). 

Moreover, as displayed in Table 3, the path coefficient between attitudes towards organic foods and purchase intention is positive (0.762) and significant (<0.05). This means that the attitudes towards organic foods will positively affect the purchase intention of consumers. Therefore, it can be said that the hypothesis 1 is accepted. Furthermore, the results of the path analysis show that social factors and attitudes towards organic foods are positive (0.251) and significant (<0.05). In addition, it is confirmed by the result of the path coefficient that the relationship between health consciousness and purchase intention is positive (0.218) and significant (<0.05). Thus, it can be inferred that hypothesis 1 is supported, while novelty seeking, quality, personal gratification, and interest in organic products with regards to status were found to have no significant relationships to purchase intention (>0.05).

The results support hypothesis H1, but also confirm the findings of previous studies, which showed that attention to health and to the environment are most frequently stated as the determinants for a positive attitude towards buying organic food [14,15,17]. On the one hand, the results show that social factors have a negative effect on attitudes towards organic foods. This means that people who rely on the expert opinions of others would view organic foods less favorably. On the other hand, the study also found that social factors have a positive effect on the purchase intention. This might be true as to a certain extent, people in Romania under normative influences will probably purchase organic foods in their effort to impress others. The findings show that being conscious of value has a positive effect on attitudes towards organic foods, with this also having a significant positive relationship to the purchase intention of organic foods. 

The results also show that quality has a significant positive effect on attitudes towards organic foods. This is because people appreciate the originality of a product and they are also aware of the social benefits if they consume organic foods. It is proven by the results of the study that personal gratification has a positive effect on attitudes towards organic foods, even if it has no significant relationship to the purchase intention of organic foods. 

Hypothesis H2 predicts that the views of product prices negatively affect the attitude of the purchase of organic food. With regards to this hypothesis, this study found that the probability of buying products with status had no significant relationship with both the attitude towards organic foods and the purchase intention of organic foods. The logic is that people who are concerned with their status will not prefer to purchase organic foods [43,44]. Apparently, their attitudes towards organic food have a positive effect on the purchase intention of organic foods. The attitude of consumers towards organic food plays an important role in leading or driving the purchase intention of consumers.

There are some reasons that can explain why some of our hypotheses were not supported. First, the beliefs of customers about the organic product itself can influence their attitudes not only towards the products, but also towards the whole experience of the trial (i.e., shopping). Second, the results show that most of their effort is spent on understanding the organic products, and they do not pay as much attention to environmental concerns.

**Figure 1:**
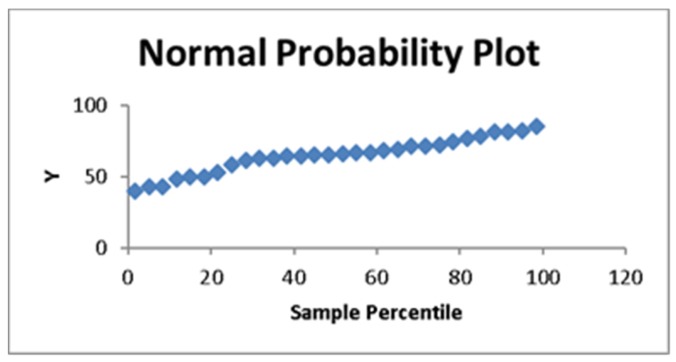
Confidence in the linear model.

The results of this study refute the statistical hypothesis that all six variables should be equal. This means that at least one of the variables is different. Therefore, the F test indicates that the obtained model is a linear model, so the regression is fit for the purpose of forecasting. The analysis and interpretation of the data obtained in the linear model show that the various tests can also be described in terms of the appropriate sample multiple correlation coefficients. These coefficients help to assess the contributions of the independent variable X to the value of the dependent variable Y. Figure 1 shows that the confidence in the model increases almost linearly from 40%, reaching nearly 90% when the observations are considered.

It should be noted from this study that the importance of food marketing for successful business is widely recognized, with results from specific actions taken in Romania being below expectations. While there is no doubt that organic food is more “natural” than conventional food, social and ethical concerns are formed more on the basis of the attitudes of Romanian consumers regarding organic food.

## 5. Conclusions 

The sustainable agriculture and organic food sectors are currently experiencing rapid growth. It is important that the consumer becomes educated about both the benefits and possible risks of purchasing either non-organic or organic foods. For better protection of consumers, organic certification programs have been created in many parts of the world in order to develop a suitable label for organic food.

After analyzing the data, the antecedents of social and personality factors mostly have significant effects on the attitudes of Romanian consumers towards organic foods. The factors derived from personality factors all have a significant effect on attitudes towards organic foods. There was no relationship between attitudes towards organic foods and the probability of buying products with status. The results of the present study confirm the use of Ajzen’s Theory of Planned Behavior [35,45] in explaining the choices made by organic foods consumers. Therefore, it can be concluded that social and personality factors affect the attitudes of consumers towards organic foods. Similarly, the result confirms that there is a significant effect of the attitudes towards organic foods on purchase intention for organic foods. This can be inferred from the fact that consumer attitudes towards organic foods will positively influence the consumer to purchase organic foods, and it provides an explanation for designing interventions for changing behavior.

In contrast, the path coefficient shows that there is only a slightly significant connection of social and personality factors with purchase intention towards organic foods. Thus, it can be stated that social and personality factors have no or less considerable effects on purchase intention. The data used is topical, refers to issues addressed in addition to having representativeness and utility value for analytical approach. 

In conclusion, this paper provides not only theoretical approaches, but also suggests opportunities that the new economic context offers for Romanian organic food producers, retailers, and distributors. In order to increase the beneficial effects of organic foods on the health of the population, the following recommendations can be made: increase the consumption of organic food products and decrease foods high in solid fats with other lower-calorie or high-aromatic oils.

It is obvious that an individual’s food-related personal traits play an important role in developing purchase intention towards organic foods. The findings of the current research indicated that producers, retailers, and distributors should prioritize the market segmentation based on gender in order to make the marketing of organic food products appropriate to the target. However, the problem of the current perspective of the organic food businesses in Romania is not resolved by attempts to lower the price of these types of products, but should be solved by providing more knowledge and education on health consciousness.

It is difficult to generalize the results, but this research contains elements that have the potential to open doors for future research. It is important to understand the additional social and economic factors that could promote organic foods for healthy eating, with further analysis of the above factors possibly providing a more comprehensive picture of related behavioral intentions to buy organic food in Romania. Apparently, Romania should conduct more studies in consumers regarding organic food and carefully examine the market barriers before implementing a new strategy. The management of food companies focusing on issues at the operational level (particularly those related to receipts, payments, supply, and sales) reduces time for strategic reflections. This is why this business will not be affected in the long-term—at least as long as the consumers meet their needs and pay the price for quality.

As with other studies, there are some limitations to this paper. First, marketing of organic food can sometimes lead to negative feelings or evaluations, if it is not designed appropriately. Second, this study cannot be extended to the international level because the effect of perceived behavioral control on purchase intention of organic food varies in different cultures.

Despite all the inherent limitations, there is evidence available in relation to organic food and human health in a more generalized sense, with the main characteristic of the organic food business being sustainability.

## Figures and Tables

**Table 1:** Items and α coefficients of the constructs.

Variable Measured	Number of Items	α
Purchase intention	5	0.771
Health consciousness	4	0.900
Novelty seeking	4	0.854
Product quality	4	0.796
Personal gratification	5	0.826
Probability to pay more for a product with status	5	0.791
Attitudes towards organic foods	7	0.940

Source: Primary data (processed).

**Table 2:** Characteristics of the participants.

	Number	%
Gender		
Female	398	59.23
Male	274	40.77
Total	672	100
Age		
18–20	193	28.72
21–30	143	21.28
31–40	175	26.04
41–50	161	23.96
Total	672	100
Missing	51	

Source: Primary data (processed).

**Table 3:** Regression to determinants of purchase intention.

Variable	Path Coefficient	*t*-Value	Significant
Social factors	0.251	3.632	0.000
Health consciousness	0.218	3.513	0.001
Novelty seeking	0.030	0.470	0.639
Product quality	0.119	1.728	0.085
Personal gratification	−0.056	−0.928	0.355
Probability to pay more for a product with status	−0.064	−0.807	0.420
Attitudes towards organic foods	0.762	6.944	0.000
	adjusted *R*^2^ = 0.622		F= 59.556
	*R* = 0.795		Sig. = 0.000

Notes: dependent variable = attitudes towards organic foods; significant at α = 5%; Source: Primary data (processed).
